# Adipocytes as an Important Source of Serum S100B and Possible Roles of This Protein in Adipose Tissue

**DOI:** 10.1155/2010/790431

**Published:** 2010-06-28

**Authors:** Carlos Alberto Gonçalves, Marina Concli Leite, Maria Cristina Guerra

**Affiliations:** Departamento de Bioquímica, Instituto de Ciências Básicas da Saúde, Universidade Federal do Rio Grande do Sul, Ramiro Barcelos, 2600-Anexo, 90035-003 Porto Alegre, RS, Brazil

## Abstract

Adipocytes contain high levels of S100B and *in vitro* assays indicate a modulated secretion of this protein by hormones that regulate lipolysis, such as glucagon, adrenaline, and insulin. A connection between lipolysis and S100B release has been proposed but definitive evidence is lacking. Although the biological significance of extracellular S100B from adipose tissue is still unclear, it is likely that this tissue might be an important source of serum S100B in situations related, or not, to brain damage. Current knowledge does not preclude the use of this protein in serum as a marker of brain injury or astroglial activation, but caution is recommended when discussing the significance of changes in serum levels where S100B may function as an adipokine, a neurotrophic cytokine, or an alarmin.

## 1. Introduction

Searching for peripheral molecular markers for brain damage and/or dysfunction, S100B protein appears to be a promising candidate [1–6]. In fact, studies in serum samples, after acute brain injury, show that S100B levels change; however the interpretation of results is complex, particularly because extracerebral sources contribute to the serum S100B content. Herein, we intend to discuss S100B from adipocytes as a source for the serum content of this protein and to compare the release of S100B from adipocytes with that of astrocytes, the major S100B-containing compartment in the central nervous system. We hope that this paper can contribute to the search for the biological role(s) of this protein in adipose tissue, as well as to help to understand how variations in the serum content of S100B affect physiological and pathological conditions.

## 2. Adipocytes

It is important to take into consideration some points about adipocytes, which are the target cells of this paper. Adipocytes are the main cell type in adipose tissue, which is distributed in three major anatomical areas: subcutaneous, dermal and intraperitoneal [7]. The population of adipocytes is heterogeneous in each area and subarea, based on their size and proliferative capacity, and also may be variable, depending on the region's blood flow and innervation density. In addition to adipocytes, adipose tissue contains stromal-vascular cells and immune cells and the traditional concept that tissue is a simple lipid store is no longer valid [8]. In fact, some adipocyte-derived proteins are messengers, acting on specific receptors found in endothelial cells, muscle cells, cardiomyocytes and neurons and disorders of communication between these cells are associated, for example, with diabetes and cardiovascular disorders [8, 9].

## 3. S100B in Adipocytes

S100B was initially described as a neuron-specific protein [10], but subsequent characterization revealed that this protein, in the central nervous system (CNS), is mainly localized in GFAP positive glial cells [11]. However, in the CNS, this protein was also abundantly detected in oligodendrocytes [12], in microglia [13] and even in cholinergic neurons of the hindbrain [14]. Moreover, it was also found among various cells of non-neural tissues [15, 16], such as adipocytes [17], chondrocytes [18] and melanoma cells [19]. 

 The first evidence of S100B in adipose tissue was independently obtained by Hidaka and coworkers, in Japan [20] and by Michetti and coworkers, in Italy [17]. Hidaka et al. found elevated levels of S100B in brain tissue, using a polyclonal anti-S100B and Michetti et al. found that S-100 protein in adipose tissue was comparable to that measured in the brain tissue, but that possibly S100A_1_ was also present in the preparation. Regardless of this, these studies contribute to consolidate the view that S100B exists in non-nervous tissues [18, 19]. 

 The mRNA expression of S100B was demonstrated later in adipose tissue [21]. It is important to mention that a direct correlation between the mRNA and protein levels was not observed. This absence of correlation also was observed in brain and adipose tissue in rats exposed to streptozotocin, a drug used to induce type 1 diabetes [22]. These data suggest a complex and cell-specific mechanism of S100B expression [23]. 

 Preliminary results from Guaragna in our laboratory indicate that human adipose tissue also expresses high levels of S100B and that this amount varies depending on the anatomical area of adipose tissue. In rats, we found an elevated content of S100B in rat white adipose epididymal tissue (about 1.5 ng/*μ*g of protein), comparable to that of the hippocampal tissue [24], but sample delipidation is a necessary experimental procedure to avoid an underestimation of S100B content in adipose tissue [25]. Moreover, both adipose and brain tissues increase S100B protein content when exposed to streptozotocin in diabetes or dementia models [22, 26].

## 4. Is S100B Released by Adipocytes?

There is clear evidence that adipocytes release S100B in different cell preparations, including epidydimal fat pad and freshly-isolated adipocytes (Table 1). Based on cell integrity assay (lactate dehydrogenase release), the mechanism of S100B release from adipocytes should be appropriately referred to as S100B secretion, as occurs in astrocytes and differs, for example, from S100B release from melanoma cells [34]. A positive regulation of S100B secretion in adipocytes was observed in response to glucagon and catecholamines, likely triggered by a cAMP-mediated pathway, as occurs in astrocytes [35]. Insulin, which attenuates the cAMP pathway in adipocytes, caused a decrease in S100B release [31]. 

 Nevertheless, it is important to evaluate whether this secretion could affect serum S100B levels. In other words, should variations in amount and activity of adipose tissue be considered in the interpretation of serum S100B levels? Human Brain tissue corresponds to 2% of weight body, while adipose tissue corresponds, in normal individuals, to 9–18% in men and 14–28% in women [7]. Thus, considering the amount and the *in vitro* secretion rate of S100B from the adipose tissue, it is reasonable to assume that this tissue is an important source of serum S100B that is even more important than brain tissue. 

Some evidence to support this hypothesis arose during physical exercise conditioning studies, where serum S100B elevations, putatively, were not associated with brain damage; such exercise included swimming racing [36], marathon running [37] and playing basketball [38]. The suspicion that body weight could affect serum S100B levels was examined in a study in bipolar patients [39]. Two independent studies in anorexic patients indicate a relationship between serum S100B and body weight [40, 41]. However, an appropriate study about this issue was performed only more recently [42]. Steiner's group correlated mass body index (BMI), serum levels of S100B and two well-characterized adipose-derived proteins: leptin and adipocyte-type fatty acid-binding protein (A-FABP) in individuals without a prior history of neurological or psychiatric disorders. They observed that S100B levels were closely correlated with the body mass index, as well as levels of leptin and A-FABP. 

 In support of these findings, we investigated the levels of serum and CSF S100B in 48-h-fasting Wistar rats [33]. A significant (more than two-fold) increase in serum S100B levels was observed in these rats, without changes in cerebrospinal fluid S100B. These data are in agreement with *in vitro* hormonal changes induced in the adipose tissue, under stressing conditions, and suggest that S100B release from adipocytes might be linked to lipolysis, but definitive evidence is lacking.

## 5. Is There a Role for S100B in the Energy Metabolism of Adipocytes?

**Figure 1:**
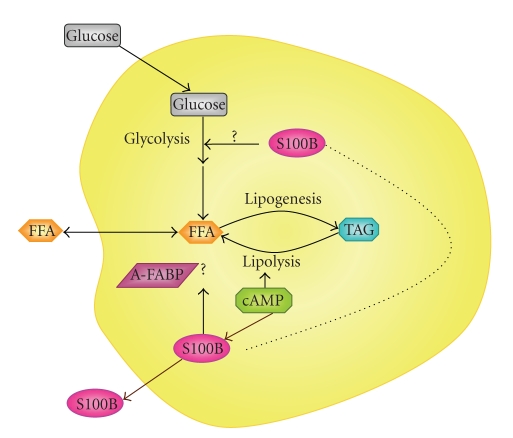
*Schematic representation of the putative roles of S100B and its secretion in adipocytes*. cAMP (e.g., induced by catecholamines—see Table 1) would trigger lipolysis and S100B secretion. The connection between these events remains to be established, as well as a role of S100B in the modulation of glycolysis (?) and in the transport of free fatty acids (?). A-FABP, adipocyte type—fatty acid binding protein; FFA, free fatty acids; TGA, triacylglycerol.

Adipocytes, like other cells, have an intense glycolytic metabolism (Figure 1). In the fed state, glucose intake goes to fatty acids and then to triacylglycerol (lipogenesis). It is important to mention that, in humans, fatty acid synthesis occurs mainly in hepatocytes. Then fatty acids, converted to triacylglycerols, are transferred and stored in adipose tissue. On the other hand, triacylglycerol stores are mobilized and brokendown to fatty acids and glycerol (lipolysis). In fact, a permanent cycle of lipogenesis and lipolysis occurs in adipose tissue, where about 70% of fatty acids released during lipolysis are reesterified. However, because adipocytes lack glycerol kinase, the glycolytic pathway provides glycerol-phosphate to lipogenesis; in addition and importantly, precursors of glucose, for example lactate (via pyruvate), are used as a source of glycerol-phosphate in a pathway denominated glyceroneogenesis (see [43] for a review). 

 Based on protein-binding assays, at least three putative targets of S100B have been implicated in energy metabolism: phosphoglucomutase [44], fructose-1,6-bisphosphate aldolase [45] and glyceraldeyde 3-phosphate dehydrogenase [23]. Apparently, S100B may inhibit phosphoglucomutase and stimulate aldolase. Together, these effects lead to an increase in the glycolytic pathway in adipocytes, which is coupled to lipogenesis and reesterification. However, the *in vitro* effects on the activity of these enzymes were preliminary characterized 20 years ago by Zimmer et al. and additional information is missing. Thus, until now a direct role of intracellular S100B on glucose metabolism in adipocytes (and astrocytes) remains a speculation. 

 Moreover, the cAMP pathway, triggered by adrenaline and glucagon (and attenuated by insulin), which results in lipolysis (see Table 1), could, in parallel, be involved in S100B release (Figure 1). In fact, cAMP modulates S100B secretion in adipocytes [27, 31] and astrocytes [35, 46]. Twenty-five years ago, Suzuki and Kato suggested that S100B could serve as a carrier protein of fatty acids [31]. However, it is not clear whether S100B release is connected to the mechanism of lipolysis induced by cAMP. It is important to mention that some members of the S100 family, such as S100A7, A8 and A9, modulate fatty acid transport in keratinocytes and neutrophils due to the interaction with fatty acid-binding proteins (FABP) [47, 48]. Interestingly, Steiner et al. observed a strong correlation between serum circulating levels of S100B and adipocyte-type FABP (A-FABP) [42]. So far, however, no study has investigated a biochemical interaction between S100B and A-FABP.

## 6. What Is the Role of S100B Release by Adipocytes?

Regardless of whether S100B is released, or not, from adipocytes during lipolysis, it is also important to evaluate the extracellular role of this protein, that is, could have this protein an autocrine, a paracrine or an endocrine role? As yet, RAGE—the Receptor for Advanced Glycation End products (AGE)—is the only characterized receptor for S100B [49, 50] and, thus, we should discuss the possible extracellular effects of adipocyte-derived S100B based on RAGE activation. However, effects of S100B, independent of RAGE, cannot be rule out (e.g., [13]). Moreover, the effect of RAGE activation depends upon ligand, that is, RAGE activation by BSA-AGE or S100B does not necessarily induce the same response [51, 52]. The diversity of effects induced by RAGE activation are not only due to cell-specificity, but also depend on the oligomeric organization of the ligand (in the case of S100 proteins), as well as RAGE oligomerization [53, 54].

 RAGE activation by AGE in cultures of adipocytes inhibited glucose uptake through the overgeneration of intracellular reactive oxygen species [55] and this could contribute to insulin resistance. Notice that *in vitro* S100B release by adipocytes is even higher than that of astrocytes [25]. Thus, beneficial or detrimental autocrine effects could be conceived. However, the direct effect of S100B on adipocytes has not been investigated until now. 

 Locally concentrated extracellular S100B in adipose tissue could exert an effect on neighbor cells. The innate immune response could be induced by S100B, recruiting monocytes [56] and activating macrophages [57], both in a manner that depends on RAGE. Moreover, also via RAGE, S100B is potentially able to increase endothelial adherence to leucocytes [58], to reduce vasodilatation induced by acetylcholine [59] and to increase neovascular proliferation [60]. Taken together, these effects could indicate a proinflammatory effect of RAGE activation by S100B, as has been proposed [61]. This is in agreement with the idea that adipocyte-derived proteins contribute to systemic inflammatory responses (e.g., [62]). However, a specific contribution of S100B to paracrine communication in adipose tissue demands further investigation, possibly providing a target for therapeutic intervention in obesity, diabetes and cardiovascular diseases. 

 With regard to any endocrine activity of the S100B released from adipocytes, two tissue targets could be proposed: the brain and heart, but weak biochemical bases of these connections can be established. S100B is not normally expressed in the myocardium, but it is induced in the peri-infarct region and potentially modulates myocyte apoptosis (see [63] for a review). Exogenous addition of S100B to cardiac myocyte cultures is able to cause apoptosis via RAGE, at a concentration of higher than 50 nM, a level that may be achieved in the peri-infarct local [64]. This concentration is 10,000 × higher than serum S100B. Thus, based on current evidence, it is quite difficult to conceive an endocrine effect of S100B, released from adipocytes, on cardiomyocytes. Furthermore, it is difficult to hypothesize an effect of S100B from adipocytes on neurons of the CNS, as the basal levels of extracellular S100B (from astrocytes and possibly from other neural cells) surpass serum levels. Therefore, the effect of S100B in the heart and CNS depends on local expression and secretion of this protein. 

 In conclusion, S100B released by adipocytes could work as an adipokine by modulating local microcirculation and immune response. In fact, due to local activation of the immune system, some S100 proteins (including S100B) have been considered a damage-associated molecular pattern (DAMP) or alarmin [65].

## 7. Contribution of Adipose Tissue to the Serum S100B Content

Generally, serum S100B is interpreted as a reflex of brain damage or astroglial activation. This is based on some properties: (1) brain tissue contains an elevated content of this protein, particularly in the astrocytes in gray matter; (2) astrocytes secrete S100B and, in fact, high levels can be measured in cerebrospinal fluid; (3) S100B is a small and very soluble protein. However, some points should be taken into consideration. Oligodendrocytes, choroid plexus epithelium and ependymal cells contains S100B [12] and potentially contribute to S100B cerebrospinal fluid (CSF) content. The size and solubility “per se” does not assure a free traffic from astrocytes or CSF to blood. In fact, there is some evidence that elevations of S100B in cerebrospinal fluid are not necessarily accompanied by elevation in serum S100B. The S100B traffic likely demands specific transporters, and it is possible that some brain diseases allow a higher S100B efflux. 

 Many extracerebral sources of S100B may contribute to the serum content of this protein. Here, we have emphasized adipocytes, but other sources include chondrocytes and cells of the marrow bone (in case of traumatism) [66, 67] and melanoma cells [68]. In these cases, S100B release appears to involve cell lysis, rather than actual S100B secretion. Other S100B-containing cells such as lymphocytes and cardiomyocytes are unlikely to contribute to serum S100B content. 

 Adipose tissue alterations, particularly insulin resistance, appear to be involved (whether preceding or associated) in many diseases, including type 2 diabetes, cardiovascular diseases and dementia [69, 70]. In addition to insulin resistance, adipose tissue alterations are also observed in bipolar affective disorders and schizophrenic patients [71], which are accompanied by elevations in serum S100B [39, 72]. Interestingly, insulin resistance in schizophrenic patients may be closely linked to serum S100B changes [73]. In support of this observation, rats fed on a ketogenic diet, which exhibits signals of insulin resistance [60], also demonstrate elevated levels of serum S100B (D Ziegler, unpublished observation). 

 Another important aspect to be considered is the ontogeny of the S100B protein. Serum S100B levels are negatively correlated with age [74]. This profile could be explained by the changes in S100B content in brain tissue (increase) and cerebrospinal fluid (decrease) observed during the postnatal development of rats [75]. Conversely, white adipose tissues increase with age [7]. This developmental characteristic apparently contradicts arguments regarding the contribution of adipocytes to serum S100B content. However, developmental evaluation of S100B content and secretion in white adipose tissue has, so far, not been investigated. Moreover, the contribution of brown adipose tissue, which contains S100B [76] and a decrease in an age-dependent manner [77], also remains to be characterized. 

 In conclusion, adipocytes contain high levels of S100B and *in vitro* assays indicate a modulated secretion of this protein by hormones that regulate lipolysis, such as glucagon, adrenaline and insulin. Although the biological significance of extracellular S100B from adipose tissue is still unclear, it is likely that this tissue might be an important source of serum S100B in situations related, or not, to brain damage. Current knowledge does not preclude the use of this protein in serum as a marker of brain injury or astroglial activation, but caution is recommended when discussing the significance of changes in its serum levels, where S100B could function as an adipokine, a neurotrophic cytokine or an alarmin.

## Figures and Tables

**Table 1:** Evidence of modulated S100B release in adipocytes.

Modulatory Agent	Effect	Preparation	Reference
Catecholamines	↑ release	epididymal fat pads	[27]
Catecholamines	↓ intracellular content	*in vivo* (adipose tissue)	[28, 29]
Epinephrine, ACTH and cAMP	↑ release	epididymal fat pads isolated adipocytes	[30]
Insulin	↓ release	epididymal fat pads	[31]
Free fatty acids	↑ release	epididymal fat pads	[32]
Epinephrine	↑ release	isolated adipocytes	[33]
	↑ basal release	isolated adipocytes	[25]

## References

[1] Bloomfield SM, McKinney J, Smith L, Brisman J (2007). Reliability of S100B in predicting severity of central nervous system injury. *Neurocritical Care*.

[2] Sen J, Belli A (2007). S100B in neuropathologic states: the CRP of the brain?. *Journal of Neuroscience Research*.

[3] Rothermundt M, Peters M, Prehn JH, Arolt V (2003). S100B in brain damage and neurodegeneration. *Microscopy Research and Technique*.

[4] Dassan P, Keir G, Brown MM (2009). Criteria for a clinically informative serum biomarker in acute ischaemic stroke: a review of S100B. *Cerebrovascular Diseases*.

[5] Gonçalves C-A, Concli Leite M, Nardin P (2008). Biological and methodological features of the measurement of S100B, a putative marker of brain injury. *Clinical Biochemistry*.

[6] Kleindienst A, Bullock MR (2006). A critical analysis of the role of the neurotrophic protein S100B in acute brain injury. *Journal of Neurotrauma*.

[7] Hausman DB, DiGirolamo M, Bartness TJ, Hausman GJ, Martin RJ (2001). The biology of white adipocyte proliferation. *Obesity Reviews*.

[8] Kershaw EE, Flier JS (2004). Adipose tissue as an endocrine organ. *Journal of Clinical Endocrinology and Metabolism*.

[9] Blüher M (2009). Adipose tissue dysfunction in obesity. *Experimental and Clinical Endocrinology and Diabetes*.

[10] Moore BW (1965). A soluble protein characteristic of the nervous system. *Biochemical and Biophysical Research Communications*.

[11] Boyes BE, Kim SU, Lee V, Sung SC (1986). Immunohistochemical co-localization of S-100b and the glial fibrillary acidic protein in rat brain. *Neuroscience*.

[12] Steiner J, Bernstein H-G, Bielau H (2007). Evidence for a wide extra-astrocytic distribution of S100B in human brain. *BMC Neuroscience*.

[13] Adami C, Sorci G, Blasi E, Agneletti AL, Bistoni F, Donato R (2001). S100B expression in and effects on microglia. *Glia*.

[14] Yang Q, Hamberger A, Hyden H, Wang S, Stigbrand T, Haglid KG (1995). S-100*β* has a neuronal localisation in the rat hindbrain revealed by an antigen retrieval method. *Brain Research*.

[15] Haimoto H, Hosoda S, Kato K (1987). Differential distribution of immunoreactive S100-*α* and S100-*β* proteins in normal nonnervous human tissues. *Laboratory Investigation*.

[16] Zimmer DB, Van Eldik LJ (1987). Tissue distribution of rat S100*α* and S100*β* and S100-binding proteins. *American Journal of Physiology*.

[17] Michetti F, Dell’Anna E, Tiberio G, Cocchia D (1983). Immunochemical and immunocytochemical study of S-100 protein in rat adipocytes. *Brain Research*.

[18] Stefansson K, Wollmann RL, Moore BW, Arnason BGW (1982). S-100 protein in human chondrocytes. *Nature*.

[19] Cocchia D, Michetti F, Donato R (1981). Immunochemical and immuno-cytochemical localization of S-100 antigen in normal human skin. *Nature*.

[20] Hidaka H, Endo T, Kawamoto S (1983). Purification and characterization of adipose tissue S-100b protein. *Journal of Biological Chemistry*.

[21] Zimmer DB, Song W, Zimmer WE (1991). Isolation of a rat S100*α* cDNA and distribution of its mRNA in rat tissues. *Brain Research Bulletin*.

[22] Zimmer DB, Chessher J, Wilson GL, Zimmer WE (1997). S100A1 and S100B expression and target proteins in type I diabetes. *Endocrinology*.

[23] Zimmer DB, Cornwall EH, Landar A, Song W (1995). The S100 protein family: history, function, and expression. *Brain Research Bulletin*.

[24] Feoli AM, Leite MC, Tramontina AC (2008). Developmental changes in content of glial marker proteins in rats exposed to protein malnutrition. *Brain Research*.

[25] Leite MC, Galland F, Brolese G (2008). A simple, sensitive and widely applicable ELISA for S100B: methodological features of the measurement of this glial protein. *Journal of Neuroscience Methods*.

[26] Rodrigues L, Biasibetti R, Swarowsky A (2009). Hippocampal alterations in rats submitted to streptozotocin-induced dementia model are prevented by aminoguanidine. *Journal of Alzheimer’s Disease*.

[27] Suzuki F, Kato K, Nakajima T (1983). Enhancement of adipose S-100 protein release by catecholamines. *Journal of Biochemistry*.

[28] Kato K, Suzuki F, Nakajima T (1983). S-100 protein in adipose tissue. *International Journal of Biochemistry*.

[29] Suzuki F, Kato K, Nakajima T (1984). Regulation of nervous system-specific S-100 protein and enolase levels in adipose tissue by catecholamines. *Journal of Neurochemistry*.

[30] Suzuki F, Kato K, Nakajima T (1984). Hormonal regulation of adipose S-100 protein release. *Journal of Neurochemistry*.

[31] Suzuki F, Kato K (1985). Inhibition of adipose S-100 protein release by insulin. *Biochimica et Biophysica Acta*.

[32] Suzuki F, Kato K (1986). Induction of adipose S-100 protein release by free fatty acids in adipocytes. *Biochimica et Biophysica Acta*.

[33] Netto CBO, Conte S, Leite MC (2006). Serum S100B protein is increased in fasting rats. *Archives of Medical Research*.

[34] Ghanem G, Loir B, Morandini R (2001). On the release and half-life of S100B protein in the peripheral blood of melanoma patients. *International Journal of Cancer*.

[35] Pinto SS, Gottfried C, Mendez A (2000). Immunocontent and secretion of S100B in astrocyte cultures from different brain regions in relation to morphology. *FEBS Letters*.

[36] Dietrich MO, Tort AB, Schaf DV (2003). Increase in serum S100B protein level after a swimming race. *Canadian Journal of Applied Physiology*.

[37] Hasselblatt M, Mooren FC, von Ahsen N (2004). Serum S100*β* increases in marathon runners reflect extracranial release rather than glial damage. *Neurology*.

[38] Schulpis KH, Moukas M, Parthimos T, Tsakiris T, Parthimos N, Tsakiris S (2007). The effect of *α*-Tocopherol supplementation on training-induced elevation of S100B protein in sera of basketball players. *Clinical Biochemistry*.

[39] Andreazza AC, Cassini C, Rosa AR (2007). Serum S100B and antioxidant enzymes in bipolar patients. *Journal of Psychiatric Research*.

[40] Ehrlich S, Salbach-Andrae H, Weiss D (2008). S100B in underweight and weight-recovered patients with anorexia nervosa. *Psychoneuroendocrinology*.

[41] Holtkamp K, Bühren K, Ponath G (2008). Serum levels of S100B are decreased in chronic starvation and normalize with weight gain. *Journal of Neural Transmission*.

[42] Steiner J, Schiltz K, Walter M (2009). S100B serum levels are closely correlated with body mass index: an important caveat in neuropsychiatric research. *Psychoneuroendocrinology*.

[43] Reshef L, Olswang Y, Cassuto H (2003). Glyceroneogenesis and the triglyceride/fatty acid cycle. *Journal of Biological Chemistry*.

[44] Landar A, Caddell G, Chessher J, Zimmer DB (1996). Identification of an S100A1/S100B target protein: phosphoglucomutase. *Cell Calcium*.

[45] Zimmer DB, Van Eldik LJ (1986). Identification of a molecular target for the calcium-modulated protein S100. Fructose-1,6-bisphosphate aldolase. *Journal of Biological Chemistry*.

[46] Gonçalves D, Karl J, Leite M (2002). High glutamate decreases S100B secretion stimulated by serum deprivation in astrocytes. *NeuroReport*.

[47] Hagens G, Masouyé I, Augsburger E, Hotz R, Saurat J-H, Siegenthaler G (1999). Calcium-binding protein S100A7 and epidermal-type fatty acid-binding protein are associated in the cytosol of human keratinocytes. *Biochemical Journal*.

[48] Roulin K, Hagens G, Hotz R, Saurat J-H, Veerkamp JH, Siegenthaler G (1999). The fatty acid-binding heterocomplex FA-p34 formed by S100A8 and S100A9 is the major fatty acid carrier in neutrophils and translocates from the cytosol to the membrane upon stimulation. *Experimental Cell Research*.

[49] Donato R, Sorci G, Riuzzi F (2009). S100B’s double life: intracellular regulator and extracellular signal. *Biochimica et Biophysica Acta*.

[50] Leclerc E, Fritz G, Weibel M, Heizmann CW, Galichet A (2007). S100B and S100A6 differentially modulate cell survival by interacting with distinct RAGE (receptor for advanced glycation end products) immunoglobulin domains. *Journal of Biological Chemistry*.

[51] Valencia JV, Mone M, Zhang J, Weetall M, Buxton FP, Hughes TE (2004). Divergent pathways of gene expression are activated by the RAGE ligands S100b and AGE-BSA. *Diabetes*.

[52] Leclerc E, Fritz G, Vetter SW, Heizmann CW (2009). Binding of S100 proteins to RAGE: an update. *Biochimica et Biophysica Acta*.

[53] Donato R (2007). RAGE: a single receptor for several ligands and different cellular responses: the case of certain S100 proteins. *Current Molecular Medicine*.

[54] Ostendorp T, Leclerc E, Galichet A (2007). Structural and functional insights into RAGE activation by multimeric S100B. *The EMBO Journal*.

[55] Unoki H, Bujo H, Yamagishi S-I, Takeuchi M, Imaizumi T, Saito Y (2007). Advanced glycation end products attenuate cellular insulin sensitivity by increasing the generation of intracellular reactive oxygen species in adipocytes. *Diabetes Research and Clinical Practice*.

[56] Shanmugam N, Kim YS, Lanting L, Natarajan R (2003). Regulation of cyclooxygenase-2 expression in monocytes by ligation of the receptor for advanced glycation end products. *Journal of Biological Chemistry*.

[57] Esposito G, De Filippis D, Cirillo C, Sarnelli G, Cuomo R, Iuvone T (2006). The astroglial-derived S100*β* protein stimulates the expression of nitric oxide synthase in rodent macrophages through p38 MAP kinase activation. *Life Sciences*.

[58] Feng L, Matsumoto C, Schwartz A, Schmidt AM, Stern DM, Pile-Spellman J (2005). Chronic vascular inflammation in patients with type 2 diabetes: endothelial biopsy and RT-PCR analysis. *Diabetes Care*.

[59] Gao X, Zhang H, Schmidt AM, Zhang C (2008). AGE/RAGE produces endothelial dysfunction in coronary arterioles in type 2 diabetic mice. *American Journal of Physiology*.

[60] Ribeiro LC, Chittó AL, Müller AP (2008). Ketogenic diet-fed rats have increased fat mass and phosphoenolpyruvate carboxykinase activity. *Molecular Nutrition and Food Research*.

[61] Hofmann MA, Drury S, Fu C (1999). RAGE mediates a novel proinflammatory axis: a central cell surface receptor for S100/calgranulin polypeptides. *Cell*.

[62] Tilg H, Moschen AR (2006). Adipocytokines: mediators linking adipose tissue, inflammation and immunity. *Nature Reviews Immunology*.

[63] Tsoporis JN, Mohammadzadeh F, Parker TG S100B: a multifunctional role in cardiovascular pathophysiology.

[64] Tsoporis JN, Overgaard CB, Izhar S, Parker TG (2009). S100B modulates the hemodynamic response to norepinephrine stimulation. *American Journal of Hypertension*.

[65] Bianchi ME (2007). DAMPs, PAMPs and alarmins: all we need to know about danger. *Journal of Leukocyte Biology*.

[66] Andersen RE, Hansson L-O, Nilsson O, Dijlai-Merzoug R, Settergren G (2001). High serum S100b levels for trauma patients without head injuries. *Neurosurgery*.

[67] Missler U, Orlowski N, Nötzold A, Dibbelt L, Steinmeier E, Wiesmann M (2002). Early elevation of S-100B protein in blood after cardiac surgery is not a predictor of ischemic cerebral injury. *Clinica Chimica Acta*.

[68] Harpio R, Einarsson R (2004). S100 proteins as cancer biomarkers with focus on S100B in malignant melanoma. *Clinical Biochemistry*.

[69] Huffman DM, Barzilai N (2009). Role of visceral adipose tissue in aging. *Biochimica et Biophysica Acta*.

[70] Luchsinger JA, Gustafson DR (2009). Adiposity, type 2 diabetes, and Alzheimer’s disease. *Journal of Alzheimer’s Disease*.

[71] Jakovljević M, Crnčević Ž, Ljubičić D, Babić D, Topić R, Šarić M (2007). Mental disorders and metabolic syndrome: a fatamorgana or warning reality?. *Psychiatria Danubina*.

[72] Schroeter ML, Steiner J (2009). Elevated serum levels of the glial marker protein S100B are not specific for schizophrenia or mood disorders. *Molecular Psychiatry*.

[73] Steiner J, Walter M, Guest P (2010). Elevated S100B levels in schizophrenia are associated with insulin resistance. *Molecular Psychiatry*.

[74] Portela LVC, Tort ABL, Schaf DV (2002). The serum S100B concentration is age dependent. *Clinical Chemistry*.

[75] Tramontina F, Conte S, Gonçalves D (2002). Developmental changes in S100B content in brain tissue, cerebrospinal fluid, and astrocyte cultures of rats. *Cellular and Molecular Neurobiology*.

[76] Barbatelli G, Morroni M, Vinesi P, Cinti S, Michetti F (1993). S-100 protein in rat brown adipose tissue under different functional conditions: a morphological, immunocytochemical, and immunochemical study. *Experimental Cell Research*.

[77] Mattson MP (2010). Perspective: does brown fat protect against diseases of aging?. *Ageing Research Reviews*.

